# Vertebral derotation in adolescent idiopathic scoliosis causes hypokyphosis of the thoracic spine

**DOI:** 10.1186/1471-2474-13-99

**Published:** 2012-06-12

**Authors:** Kota Watanabe, Takayuki Nakamura, Akio Iwanami, Naobumi Hosogane, Takashi Tsuji, Ken Ishii, Masaya Nakamura, Yoshiaki Toyama, Kazuhiro Chiba, Morio Matsumoto

**Affiliations:** 1 Department of Advanced Therapy for Spine and Spinal Cord Disorders, Keio University, Tokyo, Japan; 2 Department of Orthopaedic Surgery, Keio University, Tokyo, Japan; 3 DePuy Japan, Johnson & Johnson KK, Tokyo, Japan

## Abstract

**Background:**

The purpose of this study was to test the hypothesis that direct vertebral derotation by pedicle screws (PS) causes hypokyphosis of the thoracic spine in adolescent idiopathic scoliosis (AIS) patients, using computer simulation.

**Methods:**

Twenty AIS patients with Lenke type 1 or 2 who underwent posterior correction surgeries using PS were included in this study. Simulated corrections of each patient’s scoliosis, as determined by the preoperative CT scan data, were performed on segmented 3D models of the whole spine. Two types of simulated extreme correction were performed: 1) complete coronal correction only (C method) and 2) complete coronal correction with complete derotation of vertebral bodies (C + D method). The kyphosis angle (T5-T12) and vertebral rotation angle at the apex were measured before and after the simulated corrections.

**Results:**

The mean kyphosis angle after the C + D method was significantly smaller than that after the C method (2.7 ± 10.0° vs. 15.0 ± 7.1°, p < 0.01). The mean preoperative apical rotation angle of 15.2 ± 5.5° was completely corrected after the C + D method (0°) and was unchanged after the C method (17.6 ± 4.2°).

**Conclusions:**

In the 3D simulation study, kyphosis was reduced after complete correction of the coronal and rotational deformity, but it was maintained after the coronal-only correction. These results proved the hypothesis that the vertebral derotation obtained by PS causes hypokyphosis of the thoracic spine.

## Background

Posterior correction and fusion surgery with a segmental pedicle screw (PS) construct has been widely utilized for the surgical treatment of patients with adolescent idiopathic scoliosis (AIS), because it allows for better curve correction in the coronal plane [1-4] and the axial planes with direct vertebral rotation (DVR), compared with the use of hook or hybrid constructs [5,6]. However, a postoperative decrease in thoracic kyphosis has been reported in association with posterior correction surgery using a PS construct [7,8]. While slight increases in thoracic kyphosis have been obtained using a hook construct [9-14], PS constructs are reported to decrease the thoracic kyphosis by 3°-14° after surgery [2,3,13,15,16]. However, previous studies have also shown that PS constructs produce a significantly better vertebral derotation effect than hook-and-rod constructs [5,6,17]. The mean derotation angle obtained with a PS construct is reported to be 7.1-13.0° [5,18,19], while it is 0.4°-3.6° [20-26] with a hook-and-rod construct and 1.9°- 4.2° with the sublaminar wiring technique [27,28]. We therefore hypothesized that the decrease in thoracic kyphosis after posterior correction surgery using PS constructs was associated with the correction of vertebral rotation. The purpose of this study was to test this hypothesis by analyzing computer-simulated corrections, using three-dimensional (3D) scoliosis models based on CT data from patients with AIS.

## Methods

Twenty consecutive AIS patients (all female) who underwent posterior correction and fusion surgeries with a segmental PS construct between November 2008 and October 2009 were included in this study. All the patients had a major thoracic curve (Lenke type 1: 15 patients; type 2: 5 patients). The mean age at the time of surgery was 15.9 ± 3.2 years (range, 12–23 years). The mean preoperative Cobb angle of the main thoracic curve was 58 ± 13° (range, 41-81°), and the mean preoperative thoracic kyphosis (T5-12) was 18.9 ± 7.5° (range, 4.1-28.8°) on standing radiographs. The 3D computer simulation was conducted for all the patients.

**Figure 1:**
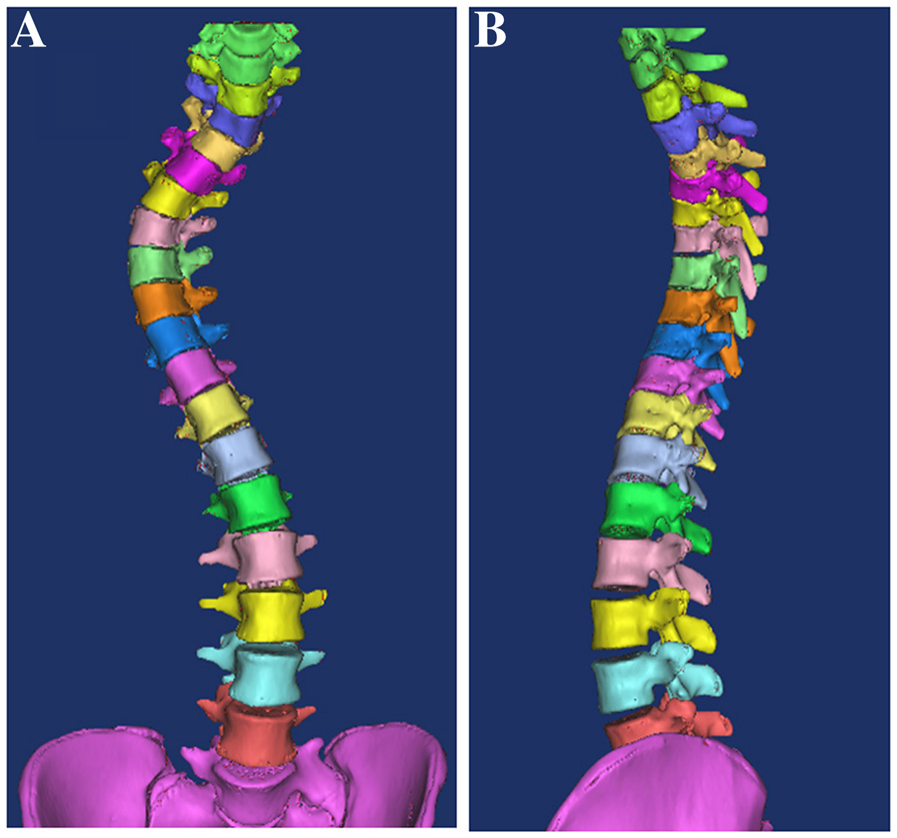
**Segmented 3D spine model.A:** Frontal view. **B:** Lateral view. A segmented 3D model of the whole spine, in which is each vertebra could be manipulated independently, was created using 3D image-processing software (Mimics; Materialise NV, Belgium), and based on the preoperative CT scan data.

The simulated corrections of scoliosis for each patient were performed on segmented 3D models of the whole scoliotic spine, created using 3D image processing software (Mimics; Materialise NV, Belgium) and based on a 1-mm-thick preoperative CT scan slice. Each segmented 3D model of the whole spine was created as follows: First, a 3D surface-reconstruction model of the whole spine was created from the CT data (Image 1). Then, the L5 vertebra was extracted from Image 1 using the range of interest (ROI) edit function of the software, to create a segmented L5 vertebra. The same procedure was repeated for each vertebra from L5 to T1 to create a segmented 3D model of the whole spine (Figure 1). In the segmented 3D spine model, each vertebra could be manipulated independently. The simulated correction was then performed in two different ways: 1) complete coronal correction (C correction), and 2) complete coronal correction with complete axial derotation of vertebra (C + D correction), as described below.

**Figure 2:**
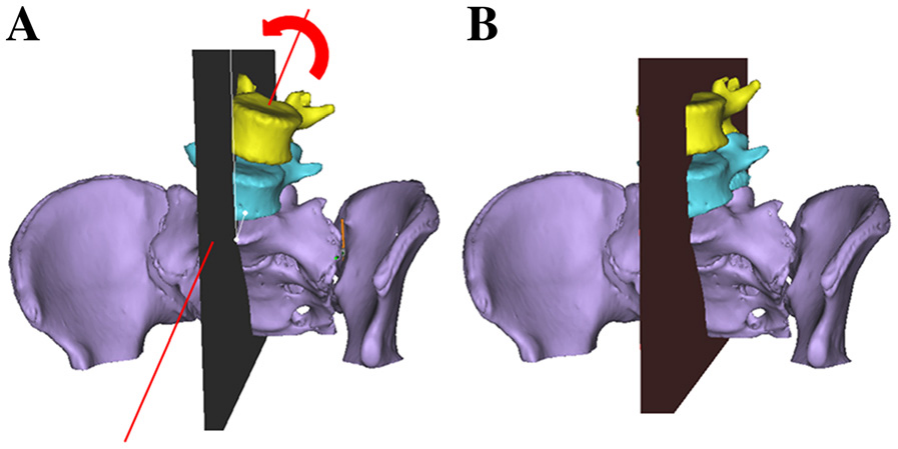
**Simulated coronal correction using the 3D segmented spine model.A:** Before correction of the L5 vertebra. **B:** After correction of the L5 vertebra. The coronal correction was performed by rotating the vertebra around an axis consisting of intersecting points between the mid-sagittal plane and the anterior and posterior margin of the upper endplate of the lower vertebra (S1), thus constraining the correction to the coronal plane (**A**). The correction was continued until the upper endplate of the L5 vertebra became horizontal (**B**).

Complete coronal correction (C correction) (Figure 2): First, the mid-sagittal plane of the whole spine was defined as the plane that included three anatomical landmark points: one at the center of the posterior vertebral wall of C7, and two at the centers of the anterior and posterior margins of the upper sacral endplate. The correction was started from the L5 vertebra and performed by rotating the L5 vertebra around the axis consisting of the intersecting points between the mid-sagittal plane and the anterior and posterior margin of the upper endplate of the lower vertebra (S1), thus constraining the correction to the coronal plane (Figure 2A). The correction was continued until the upper endplate of the L5 vertebra became horizontal (Figure 2B). During the correction, the vertebrae cranial to L5 were rotated together with the L5 vertebra, maintaining their original relative position. The same correction procedures were continued to C7, to align the whole spine on the mid-sagittal plane.

**Figure 3:**
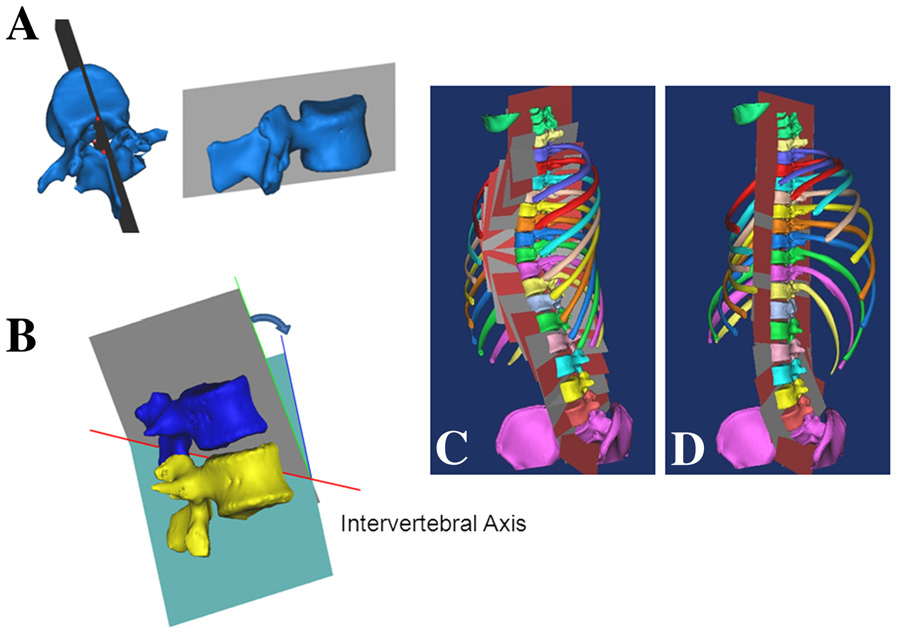
**Simulated coronal correction + derotation of vertebrae using the 3D segmented spine model.** A “reference plane” defined as a plane including three points: one at the central notch of the lamina and two at the upper and lower endplates on the posterior vertebral wall (**A**) was determined for each vertebra from T1-L5. Then, the intervertebral axis, defined as the line intersecting the neighboring reference planes, was determined (**B** and **C**). Finally, the correction of each vertebral body was performed by rotating the vertebra around the intervertebral axis until the neighboring vertebral reference planes became matched on the same plane (**B** and **D**).

Complete coronal correction with complete axial derotation of the vertebrae (C + D correction) (Figure 3): First, a “reference plane” defined as the plane including three points, one at the central notch of the lamina and two at the center of the upper and lower endplates on the posterior vertebral wall (Figure 3A), was determined for each vertebra from T1 to L5. Then, an “intervertebral axis,” defined as a line intersecting the neighboring reference planes was determined (Figure 3B). The simulated correction was performed by rotating each vertebra around the intervertebral axis until the neighboring vertebral reference planes became matched on the same plane (Figure 3C and D). The simulated correction was started at L5 and continued to T1. During the correction, the vertebrae cranial to the vertebra being corrected were rotated together with it, thus maintaining their original relative position. These corrections resulted in the complete coronal correction and axial derotation of the vertebrae (Figure 3D).

To evaluate the relationship between thoracic kyphosis and vertebral derotation, the thoracic kyphosis angle (T5-12), the radius of the thoracic curvature, and the vertebral rotation angle at the apex were measured before and after the two different simulated corrections for each patient. The thoracic kyphosis angle and radius of thoracic curvature were measured on the mid-sagittal plane. The radius of curvature at a given point is the radius of a circle that mathematically best fits the spinal curve at that point. The radius of curvature was measured at each adjacent segment from T1-T12, and then a value for the whole thoracic spine was determined as the mean of the values for each segment. The vertebral rotation angle at the apex was measured using Aaro’s method [29] against a reference point set at the pelvis. The clinical relevance of these simulated corrections was evaluated by comparing the values obtained from the simulations with those measured on the postoperative CT taken for each patient.

Surgical procedures included the segmental placement of the PS, placement of the first rod on the concave side of the curve, rod rotation maneuver for the sagittal and coronal corrections, in-situ contouring for coronal correction, direct vertebral derotation for axial correction, and placement of the second rod, as described previously [18,30,31].

This study was approved by the medical ethics committee of Keio University Hospital (2009-203-2).

## Results

**Figure 4:**
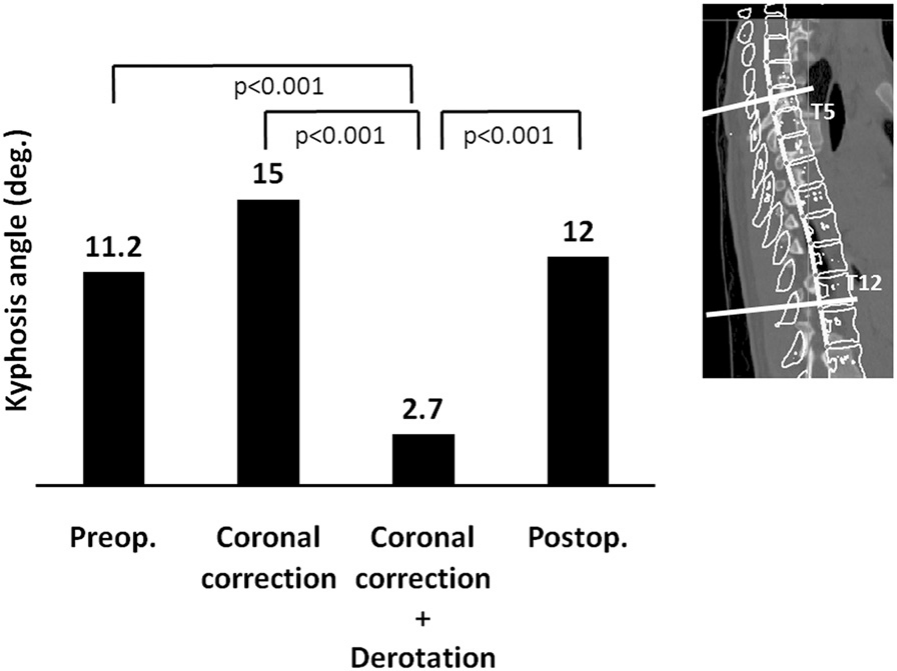
Kyphosis angle after simulated corrections and post-operative findings.

**Figure 5:**
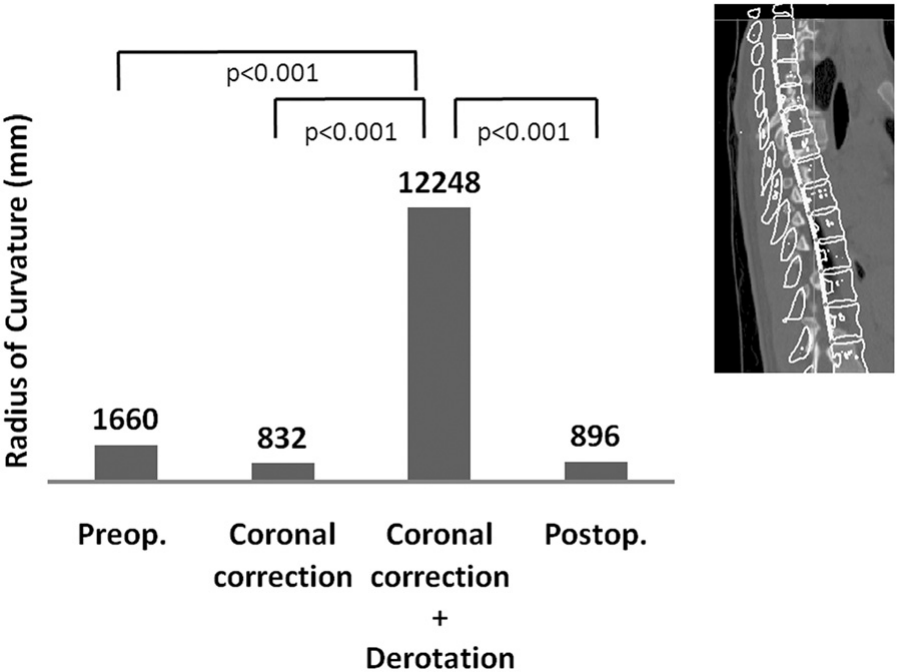
Radius of curvature after simulated corrections and post-operative findings.

**Figure 6:**
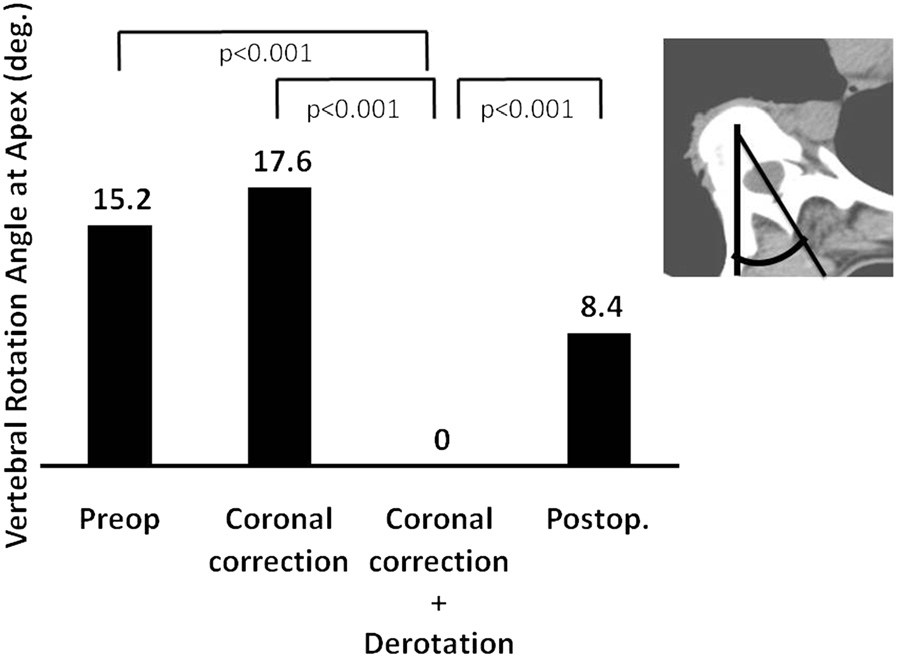
Vertebral rotation angle at the apex vertebra after simulated corrections and post-operative findings.

The mean kyphosis angle before the simulated correction was 11.2 ± 6.4, and it increased significantly to 15.0 ± 7.1, after the C correction (p = 0.02). However, the mean thoracic kyphosis angle decreased significantly to 2.7 ± 10.0 after the C + D correction (p < 0.001) (Figure 4). The mean radius of curvature of the thoracic spine before the simulated correction was 1660 ± 1461 mm (Figure 5). Although the value increased significantly, to 12248 ± 30543 mm after the C + D correction, no significant change was recognized after the C correction (832 ± 552 mm, p = 0.617). The mean radius of curvature was significantly different between the two correction methods (p < 0.001). The mean vertebral rotation angle at the apex was 15.2 ± 5.5 before the simulated correction (Figure 6), and it was 0 (completely corrected) after the C + D simulation. After the C correction, however, the mean rotation angle of the apex did not change significantly (17.6 ± 4.2, p = 0.209). The CT scans obtained after the patients’ posterior correction and fusion surgery with a segmental PS construct revealed a mean kyphosis angle of 12.0 ± 7.4 (Figure 4), a mean radius of curvature of 896 ± 373 mm (Figure 5), and a mean apical rotation angle of 8.4 ± 4.5 (Figure 6).

## Discussion

**Figure 7:**
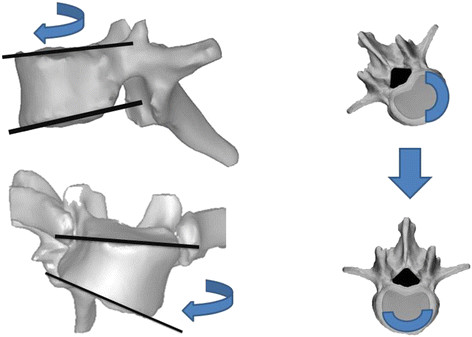
**Wedge deformity of a vertebral body in the scoliotic spine.** T9 vertebra of a 12-year-old girl with AIS. The wedged vertebra is taller at the anterior than the posterior wall, and at the convex side than the concave side. During vertebral derotation, the taller anterior and convex walls shift in the ventral direction of the spine.

The results of the 3D simulation study indicated a close relationship between vertebral derotation and a decrease in thoracic kyphosis, since the C + D simulation resulted in a significant decrease in thoracic kyphosis, while the C correction resulted in the maintenance of thoracic kyphosis. The decrease in thoracic kyphosis caused by vertebral derotation may be attributed partly to the wedge deformity of the vertebral bodies, which are taller on the convex side than on the concave side and taller ventrally than posteriorly in patients with structural scoliosis (Figure 7) [32,33]. During the correction surgery, the vertebral derotation maneuver causes both the taller convex wall and the taller anterior wall to shift in the ventral direction, thereby elongating the anterior column of the thoracic spine and ultimately resulting in a decrease in thoracic kyphosis (Figure 7).

Segmented 3D scoliosis models have been used in previous studies to reproduce actual scoliotic spine deformities and correction maneuvers, including Cotrel-Dubousset surgical maneuvers, in situ contouring, and correction with a segmental PS construct using a personalized finite element model of AIS [34-37]. The objective of these simulations was to predict the corrected spinal geometry and to assist the pre-operative planning of the surgical instrumentation to be used. However, the segmented 3D scoliosis model created in the present study eliminates the constraining effects of soft tissues, to simulate the maximum corrections in the coronal plane independent of corrections in the axial plane, which cannot be accomplished in the actual surgery. Therefore, we also evaluated the clinical relevance of this simulation model. The mean rotation angle at the apex measured on patients’ post-surgical CT images was between the mean values obtained by the simulated C correction and the simulated C + D correction (Figure 6). The mean thoracic kyphosis angle and radius of curvature were also between the mean values of the two simulated corrections (Figures 4 and 5). These results suggest that the real surgery can achieve a limited correction in the axial plane that falls between the two extremes of the simulated corrections.

## Conclusions

In conclusion, the present 3D simulation study demonstrated that the derotation of vertebrae caused a decrease in thoracic kyphosis during the correction of thoracic scoliosis.

## Consent

Written informed consent was obtained from the parents of the patient for publication of this case report and any accompanying images. A copy of the written consent is available for review from the Editor-in-Chief of this journal.

## Competing interests

The authors declare that they have no competing interests.

## Authors’ contributions

KW, MM and TN made substantial contributions to the conception and design, and the acquisition, analysis, and interpretation of data. They were also involved in drafting and revising the manuscript. AI, NH, TT, KI, MN, YT, and KC contributed to the conception and design, and performed critical revision of the manuscript. All authors read and approved the final manuscript.

## Pre-publication history

The pre-publication history for this paper can be accessed here:

http://www.biomedcentral.com/1471-2474/13/99/prepub
